# [^18^F]-fluorocholine long-axial-field of view PET-CT accurately localises intrathyroid parathyroid adenoma in 5-month pregnant patient

**DOI:** 10.1007/s00259-025-07417-6

**Published:** 2025-06-19

**Authors:** Beverley F. Holman, Tamar Willson, Jane Edwards, Bruno Ferreira, Piyumi Wijewickrama, Thomas Wagner

**Affiliations:** 1 Department of Nuclear Medicine, Royal Free London NHS Foundation Trust, London, UK; 2 Radiological Physics, Royal Free London NHS Foundation Trust, London, UK; 3 Department of Endocrinology, Royal Sussex County Hospital, Brighton, UK; 4 Division of Medicine, University College London, London, UK; 5 Centre for Medical Imaging, Department of Imaging, University College London, London, UK


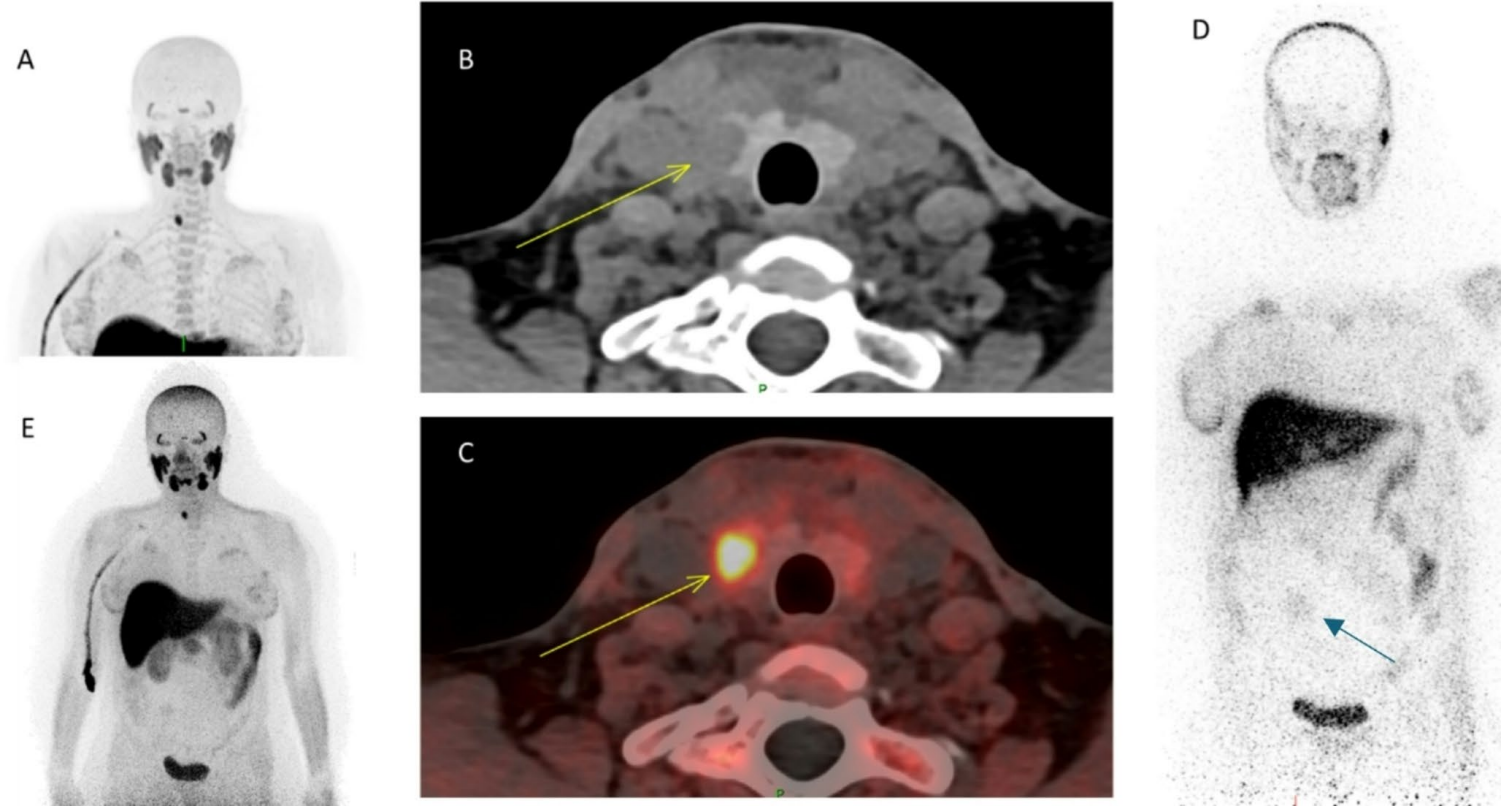
A 44-year old pregnant patient with primary hyperparathyroidism causing severe hypercalcaemia was referred for [^18^F]-fluorocholine long-axial-field of view (LAFOV) PET-CT for pre-surgical localisation of parathyroid adenoma [1]. The patient needed multiple hospitalisations and was at risk of foetal and maternal complications. Exploratory neck surgery was unsuccessful.

We performed a low-dose PET-CT to minimise radiation exposure to the foetus [2]. The patient was injected with 17 MBq (0.2 MBq/kg) and scanned for 30 min from vertex to diaphragm, 62 min post-injection using a Biograph Vision Quadra PET-CT (Siemens Healthineers, Erlangen, Germany). CT was performed with CAREDose on (reference mAs = 65), CAREkV reference 120 kV. [^18^F]-fluorocholine LAFOV PET-CT demonstrated focal intense choline uptake in a low density intrathyroid left lower pole nodule, consistent with a parathyroid adenoma (A: MIP, B: axial CT and C: fused axial images). An uncorrected PET-only acquisition was performed to calculate the foetal dose, which was estimated at 0.5 mSv from PET and 0.1 mGy from CT.

The patient had surgery the following week, which removed the parathyroid adenoma with good intraoperative parathyroid hormone drop. Pathology confirmed an intrathyroid parathyroid adenoma. The patient was discharged and was mildly hypocalcaemic following surgery.

This is the first reported case of [^18^F]-fluorocholine PET-CT in a pregnant patient. The non-attenuation corrected PET images of the patient (D: coronal view and E: MIP) demonstrated mild choline uptake, likely in the liver of the foetus. Despite choline being an essential nutrient for foetal brain development, only minimal amount of [^18^F]-fluorocholine was present in the foetus.

## Electronic supplementary material

Below is the link to the electronic supplementary material.

Supplementary Material 1
