# Supplementary material for: [18F]-fluorocholine long-axial-field of view PET-CT accurately localises intrathyroid parathyroid adenoma in 5-month pregnant patient
Source: Eur J Nucl Med Mol Imaging. 2025 Jun 19;53(4):2191–2. doi: 10.1007/s00259-025-07417-6 (PMC12920332; doi:10.1007/s00259-025-07417-6)
Supplement: Supplementary file 1 — Supplementary Material 1 [file 259_2025_7417_MOESM1_ESM.docx]

# Supplementary data file: foetal dosimetry

A vertex to pelvis PET-only image was acquired to assess the possible foetal uptake.

A nuclear medicine consultant doctor (TW) examined the image and produced a volume of interest (VOI) encompassing the foetus and likely also the amniotic fluid and uterus, using Hermes Affinity viewer.


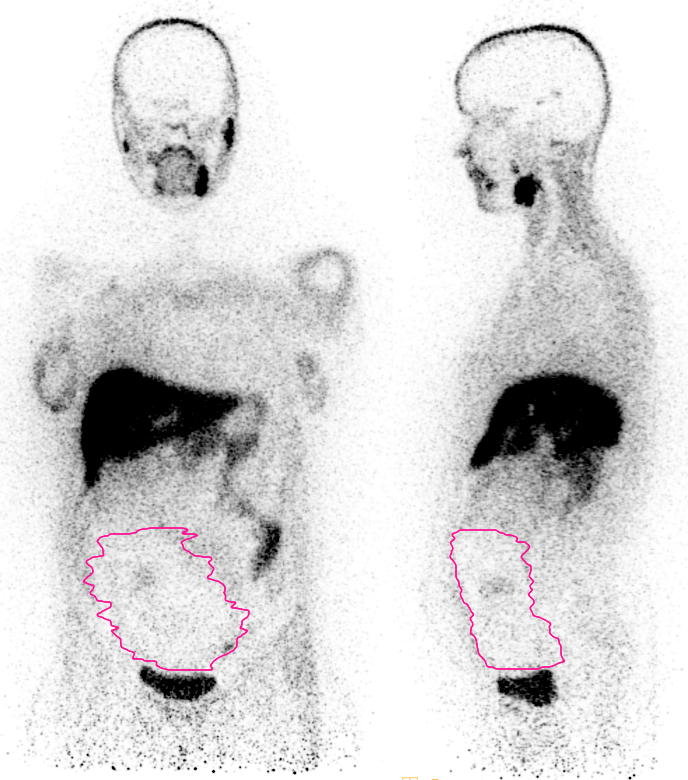
 **Figure 1: the foetal VOI outlined on the PET-only images.**

The VOI was a volume of 1.76 litres, with a mean count value of 8.2 PROPCPS. Since this is a PET-only acquisition, corrections enabling quantitative measurement of activity concentration in Bq/ml are not available. However the patient’s clinical PET/CT of the head and thorax includes attenuation correction and can be used for activity concentration measurements, and the same tissue is also visible in the PET-only acquisition. A 5 cm diameter circular region of interest was placed in the liver on both studies and the ratio of the mean values used to determine a Bq/ml/PROPCPS conversion factor. This factor is an approximation and may incur bias and uncertainty, however it allows an approximate means of estimating the activity concentration in the abdomen. Using this factor we calculated that 0.4 MBq was present in the outlined foetal VOI, decay-corrected to the injection time.

ICRP Publication 128 [1] includes factors of absorbed dose per unit of activity administered for different populations. The factor provided by the ICRP for a 10kg 1-year old child was scaled by weight to the 1.76 kg assumed mass of our foetal volume of interest, to produce a coefficient of 0.57 mSv/MBq for the foetus. This yields an absorbed dose of 0.22 mSv from placental transfer.

The foetal dose received as passive exposure from the mother’s other organs was estimated using the dose to the uterus per MBq of administered activity given in the ARSAC notes for guidance [2], yielding an absorbed dose of 0.26 mSv and so a foetal exposure of 0.48 mSv from the PET study.

Foetal dose from the CT component was calculated using the NCICT dosimetry system [3] using the appropriate gestational age phantom. Foetal dose was estimated to be less than 0.1 mGy from the CT of the neck and thorax.

1. Administration of Radioactive Substances Advisory Committee. ARSAC notes for guidance: good clinical practice in nuclear medicine. 31 March 2025. <https://www.gov.uk/government/publications/arsac-notes-for-guidance> Accessed 04/03/2025.
2. ICRP, 2015. Radiation Dose to Patients from Radiopharmaceuticals: A Compendium of Current Information Related to Frequently Used Substances. ICRP Publication 128. Ann. ICRP 44(2S).
3. NIH National Cancer Institute. NCICT: NCI dosimetry system for computed tomography. https://dceg.cancer.gov/tools/radiation-dosimetry-tools/computed-tomography
